# Cortical screw placement with a spinous process clamp guide: a cadaver study accessing accuracy

**DOI:** 10.1186/s12893-022-01829-z

**Published:** 2022-11-08

**Authors:** Xi-nuo Zhang, Yi-qi Zhang, Yu-zeng Liu, Qing-jun Su, Li Guan, Dong-yue Li, Bao-qing Pei, Ai-xing Pan, Hong-hao Yang, Hong-tao Ding, Yong Hai, Li-jin Zhou

**Affiliations:** 1 Department of Orthopedic Surgery, Beijing Chao-Yang Hospital, Capital Medical University, 8 Gong Ti Nan Lu, Chaoyang District, Beijing, 100020 China; 2 Beijing Key Laboratory for Design and Evaluation Technology of Advanced Implantable and Interventional Medical Devices, Beijing Advanced Innovation Center for Biomedical Engineering, School of Biological Science and Medical Engineering, Beihang University, Beijing, 100083 China

**Keywords:** Cortical bone trajectory, Guide, Navigation, Lumbar spine fixation, Cadaveric study

## Abstract

**Background and objective:**

The Cortical Bone Trajectory (CBT) technique provides an alternative method for fixation in the lumbar spine in patients with osteoporosis. An accuracy CBT screw placement could improve mechanical stability and reduce complication rates.

**Purpose:**

The purpose of this study is to explore the accuracy of cortical screw placement with the application of implanted spinous process clip (SPC) guide.

**Methods and materials:**

Four lumbar specimens with T12-S1 were used to access the accuracy of the cortical screw. The SPC-guided planning screws were compared to the actual inserted screws by superimposing the vertebrae and screws preoperative and postoperative CT scans. According to preoperative planning, the SPC guide was adjusted to the appropriate posture to allow the K-wire drilling along the planned trajectory. Pre and postoperative 3D-CT reconstructions was used to evaluate the screw accuracy according to Gertzbein and Robbins classification. Intraclass correlation coefficients (ICCs) and Bland–Altman plots were used to examine SPC-guided agreements for CBT screw placement.

**Results:**

A total of 48 screws were documented in the study. Clinically acceptable trajectory (grades A and B) was accessed in 100% of 48 screws in the planning screws group, and 93.8% of 48 screws in the inserted screws group (p = 0.242). The incidence of proximal facet joint violation (FJV) in the planning screws group (2.1%) was comparable to the inserted screws group (6.3%) (p = 0.617). The lateral angle and cranial angle of the planned screws (9.2 ± 1.8° and 22.8 ± 5.6°) were similar to inserted screws (9.1 ± 1.7° and 23.0 ± 5.1°, p = 0.662 and p = 0.760). Reliability evaluated by intraclass correlation coefficients and Bland–Altman showed good consistency in cranial angle and excellent results in lateral angle and distance of screw tip.

**Conclusions:**

Compared with preoperative planning screws and the actually inserted screws, the SPC guide could achieve reliable execution for cortical screw placement.

## Introduction

A cortical bone trajectory (CBT) is a novel technique providing an alternative approach for pedicle screw placement in lumbar spinal surgery [1]. The entry point of the cortical screw trajectory is the interarticular lateral part at the level of the inferior border of the transverse process, and the trajectory follows along the cranio-caudal and medial–lateral path through the pedicle [2]. The CBT technique maximizes the contact of cortical bone surface with the screw threads and enhances the fixation strength due to its cranial and lateral screw trajectory, which is able to increase the strength of the fixation screw and minimize the incidence of loosening. In addition, the characteristic of the trajectory has the advantage of avoiding extensive exposure of the cephalad facet joints and minimizing muscle dissection to provide a minimal invasive surgical procedure.

Previous studies have reported the accuracy of cortical screw placement with the assistance of 3D printing guides, 3D fluoroscopy-assisted navigation technology, and robot-assisted screw implantation [3–5]. Although 3D printed guides provide precise navigation, requirements including expensive price, consume human resources, and fully stripping of paraspinal soft tissue and facet joint capsule need to be considered [3]. Furthermore, 3D navigation and robot-assisted surgery rely on the CT scans to evaluate intraoperative screw localization which could increase the risk of radiation exposure.

This study has provided the initial experience of the application of spinous process clamp (SPC) guided cortical screw placement and evaluated the accuracy of the device. The SPC guide was a device that anchored on the spinous process of the surgical segment vertebral body through a special tooth-like clamp, providing cortical trajectories for patients requiring spinal fusion between L1 and S1. According to the preoperative 3D-CT reconstruction, planning adjusts the position and posture of the SPC guide during the procedure to make sure that the screw was inserted precisely along the cortical trajectory.

## Methods

Four formalin-treated cadaveric lumbar specimens with T12–S1 segments were obtained from the China Capital Medical University for this study. All the cadaver specimens without congenital spinal deformity, pars defect, metastatic spinal lesions, trauma, infection, and history of spinal surgeries were enrolled. Paravertebral soft tissue on the lamina, pars interarticularis, and facet joint were fully stripped, but the interspinous and supraspinous ligaments were preserved. All processed specimens were temporarily stored in a refrigerator at − 20 °C until they were warmed to room temperature for further using. A total of 48 screws were placed after the cortical trajectory planning at L1 to S1. All screw placement were performed by two spine surgeons at our center with extensive experience in CBT screw placement. Screw parameters include lateral angle (LA), cranial angle (CA) and distance between screw tip (DBST) were measured in preoperative planning and actual inserted lumbar specimens. LA was defined as the angle between the symmetry axis of the vertebral body and the axis of the cortical screws. CA was defined as the angle between the cephalad endplate line and the axis of the cortical screws, DBST was the distance between two screw tips measured from 3D CT reconstructions.

### Preoperative planning

**Fig. 1:**
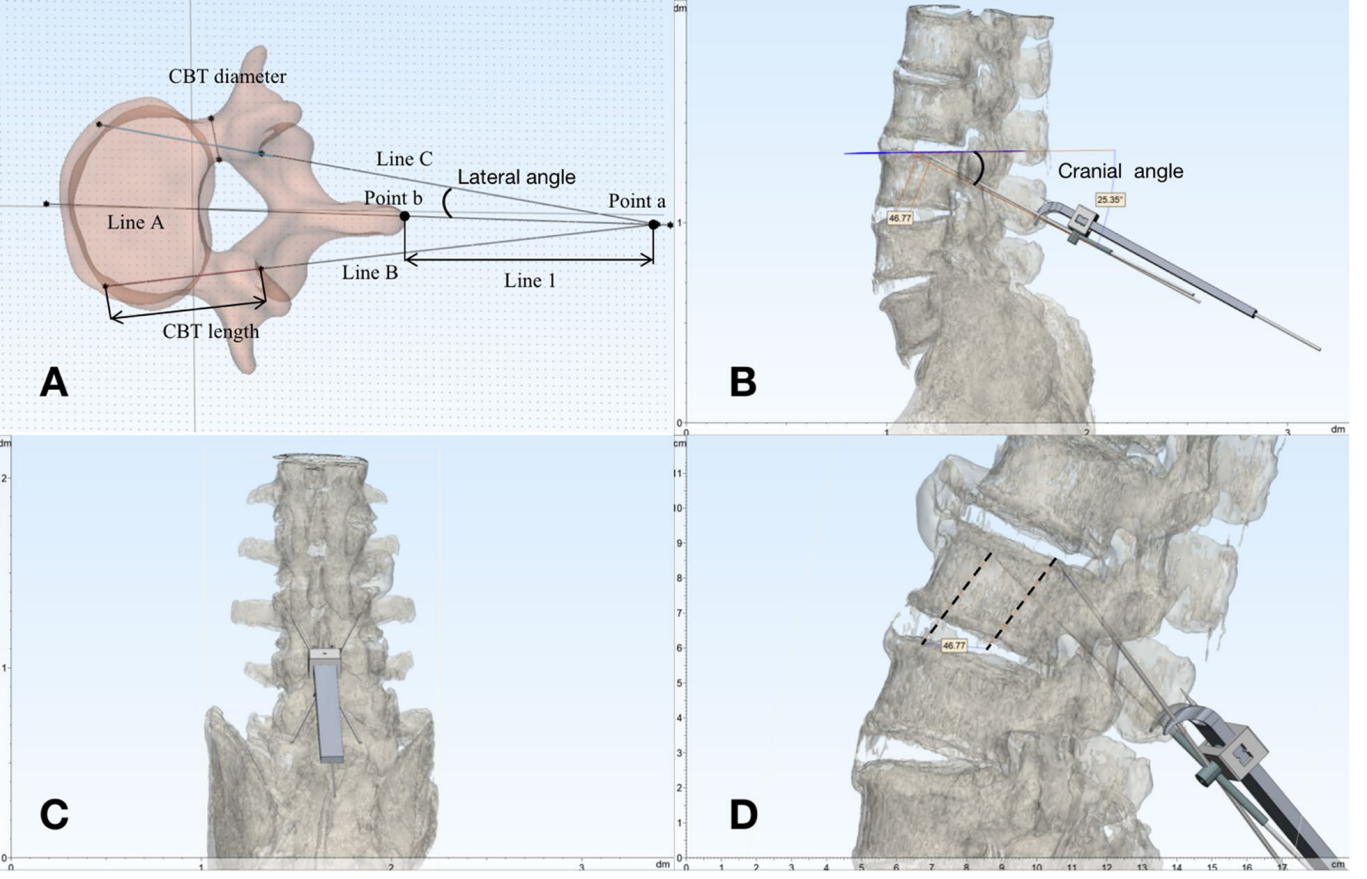
**A** the measurement parameters of SPC guide; **B** cranial angle of the planned screws guided with SPC; **C** lateral angle of the planned screws guided with SPC; **D** distance between screw tip measured in preoperative spine 3D reconstructions

The 3D-CT reconstructions with coronal, sagittal, and axial images of L1-S1 vertebrae were generated by GE Picture Archiving and Communication Software with a layer thickness of 0.625 mm voltage 120 kV, current 150 MA, and matrix 512 × 512 (PACS) (General Electric Medical Systems, Milwaukee, WI, USA). 3D lumbar models were reconstructed by Mimics^®^ (Materialise, Leuven, Belgium). The three-dimensional cortical screw trajectory planning including the starting point, CA, LA, screw length, screw diameter, and the length between the spinous process to knob was achieved by Solidworks^®^ (Dassault Sysètmes, Vélizy-Villacoublay, France) (Fig. 1).

Shown in the Fig. 1A and B, Line A is the central axis that passes through the spinous process and the vertebral body, and lines B and C are the axes of the cortical trajectory screw. In preoperative CT planning, lines A and B pass through the pedicle and the vertebral body bone as long as possible. These two lines intersect with line A at one focus. Two focuses have occurred when the spine with deformity, trauma, etc. According to the distance between the focus and the spinous process, the fixed height of the navigator knob is determined, and the scale at the root of the spinous process clip is marked as 0. The angle between line B or C and line A was defined as the lateral angle of the cortical trajectory, and the navigator knob was adjusted during operation according to the planned lateral angle. The specifications of the CBT screw are also determined according to the measurement parameters of the preoperative CT planning. The length of line B and line C inside the pedicle and the vertebral body was the CBT screw length. Compared with screw length, the diameter measurement of screws was a little more complicated. The shortest distance between line B or line C and the medial wall or lateral wall of the pedicle was the radius of the CBT screw. In case of the cortical screw cannot be placed on the screw trajectory even with a 4.5 mm diameter screw, due to the pedicle diameter being too small, to minimise the perforation area is a plan being made.

### Application of spinous process clamps (SPC) guide

**Fig. 2:**
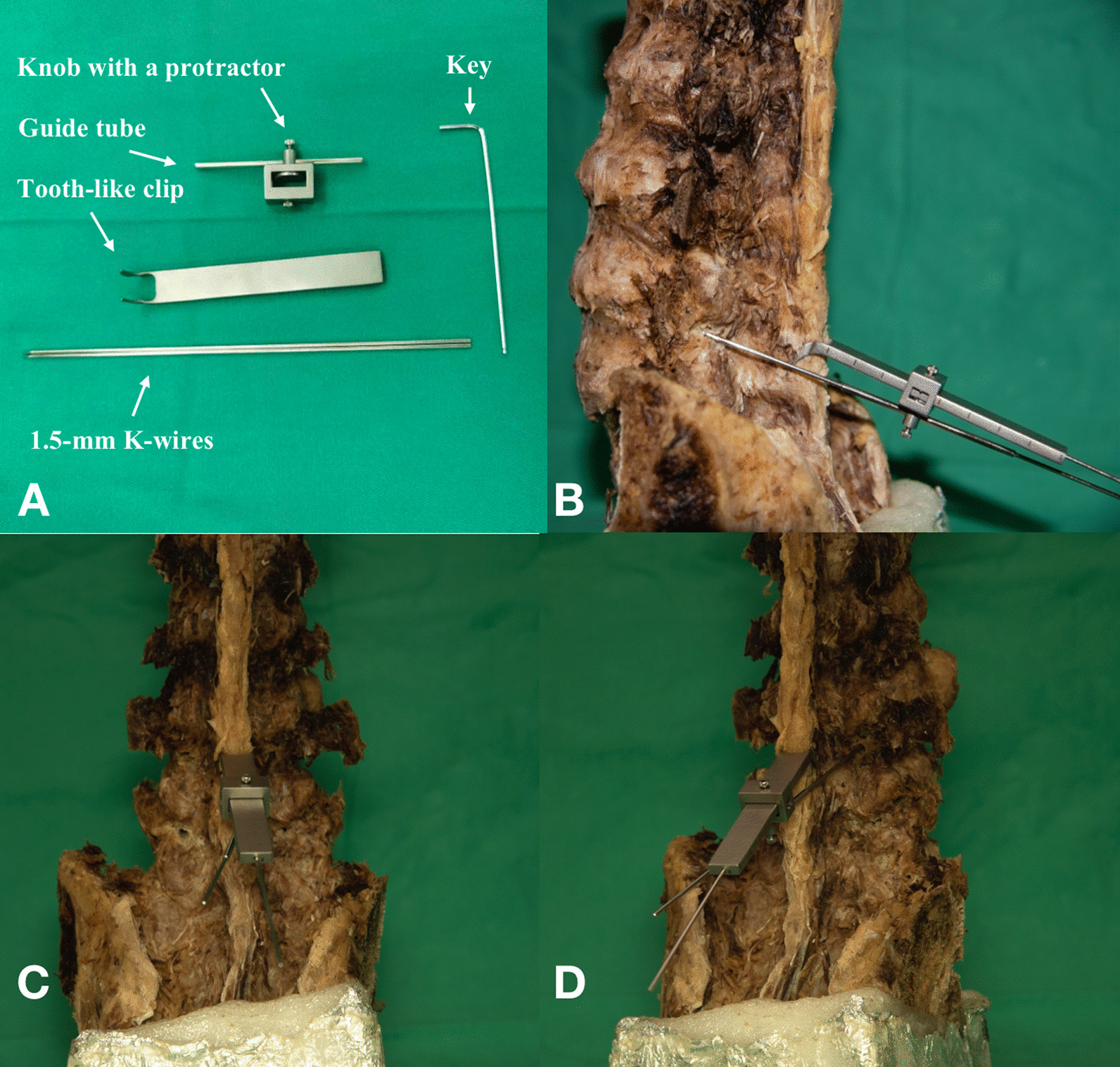
**A** Hardware of SPC guide; **B** the SPC guide was anchored on the specimen in sagittal view; **C** the SPC guide was anchored on the specimen in coronal view; **D** the SPC guide was anchored on the specimen in post-lateral view

**Fig. 3:**
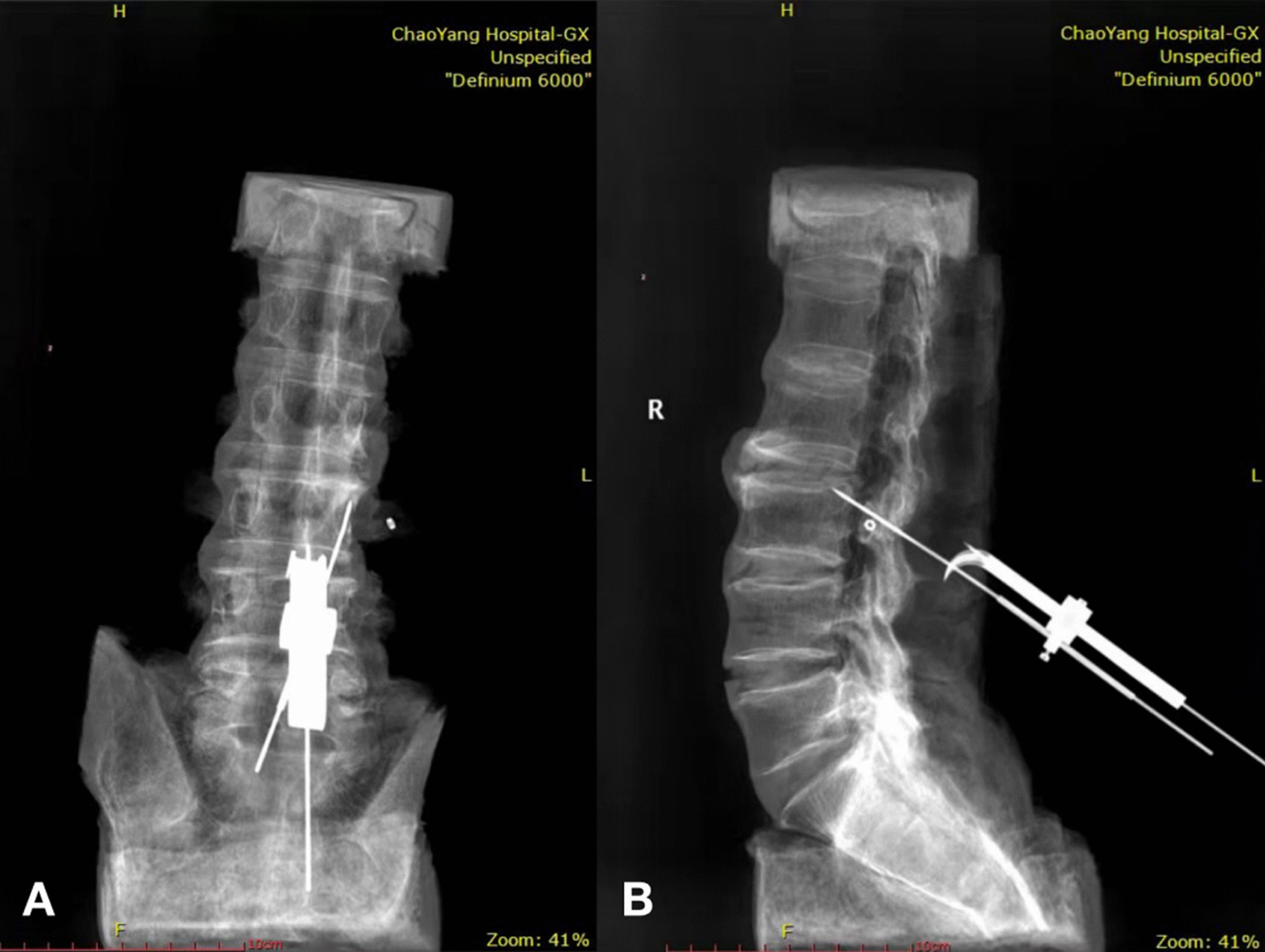
**A** The SPC guide was anchored on the specimen in anteroposterior imaging; **B** the SPC guide was anchored on the specimen in sagittal imaging

As shown in Fig. 2, the SPC guide is a navigation device that is rigidly attached to the bone tissue and consists of a spinous process with a curved tooth-like clip, a knob with a protractor, a guide tube, and two 1.5 mm K-wires. After the SPC guide was installed on the spinous process, the caudo-cephalad angle of the device was adjusted based on preoperative planning and locked by 1.5-mm K-wires. Laterally X-ray was used to check the caudo-cephalad angle of the SPC guide. The knob was locked at a height planned before the operation. The lateral angle was adjusted to the angle planned preoperative, and the second 1.5 mm K-wire was anchored on the par through the guide tube. Anteroposterior imaging to confirm the starting point of cortical trajectory was at the medial inferior edge of pedicle projection. A K-wire or drill was used to created drill holes after the starting point was confirmed, and a 3.5 mm bit was used to drill a complete path (Fig. 3). Finally, the burr allows for tactile feedback through the pedicle.

### Assessment

**Fig. 4:**
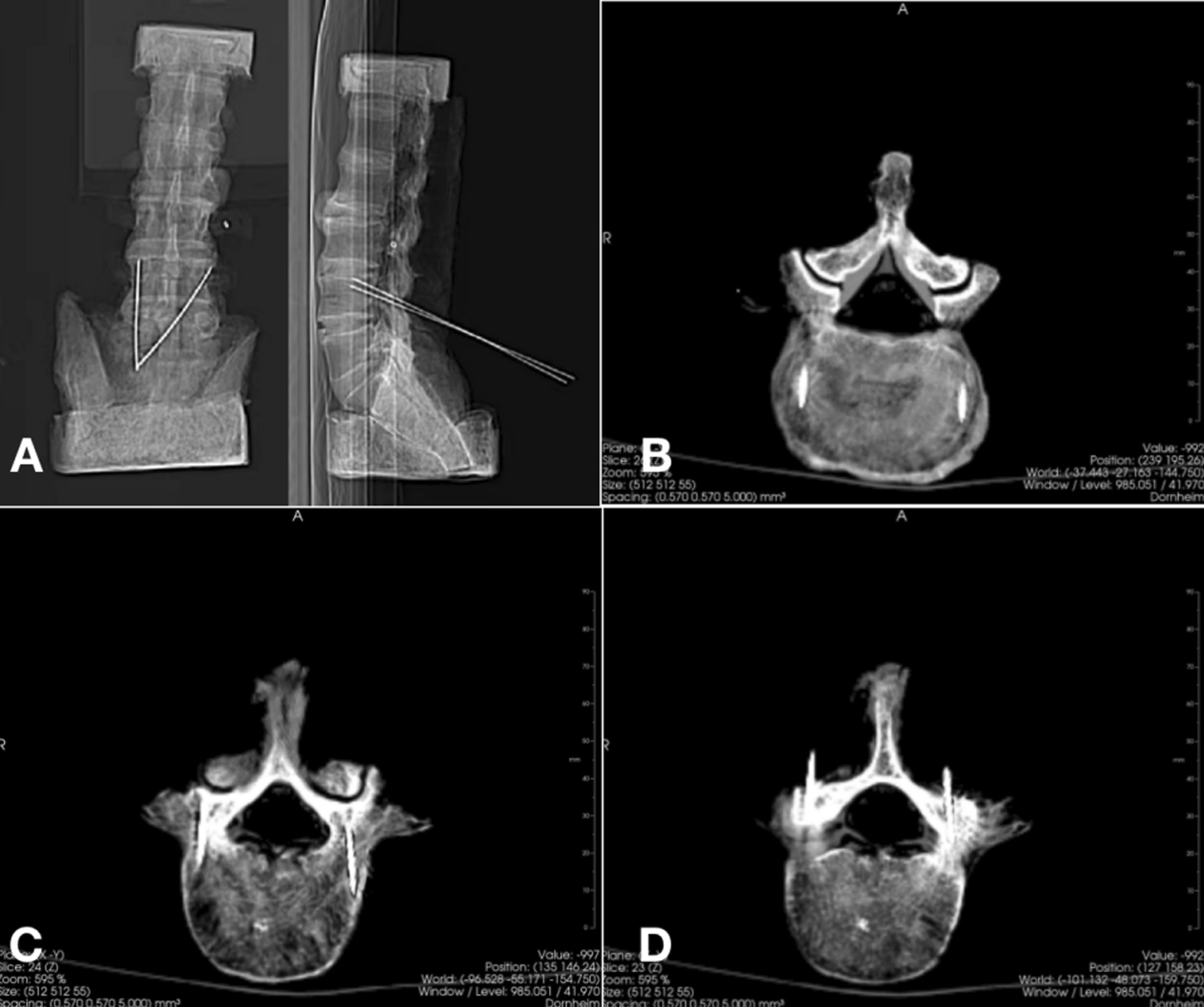
**A** CT scan location image; **B** the first layer of 1.5 mm K-wires in L4 vertebrae; **C** the second layer of 1.5 mm K-wires in L4 vertebrae and pedicle; **D** the third layer of 1.5 mm K-wires in L4 pedicle

3D reconstructions of vertebrae and insert screws were established from postoperative CT scans (Fig. 4). Preoperative and postoperative 3D-CT reconstructed vertebrae and screws was superimposed and measured the parameters of any deviation of inserted screws from planned. The deviation of the CBT screw from the pedicle to the vertebral body was measured in millimeters. The accuracy of cortical screw placement was assessed using the modified method of Gertzbein and Robbins (Grade A, no perforation; Grade B, 0–2 mm; Grade C, 2–4 mm; Grade D, 4–6 mm; Grade E > 6 mm) [3, 6]. Clinically acceptable trajectories correspond to grades A and B, while deviations worse than grade C are considered unacceptable [4]. Axial and sagittal planes from reconstructed CT scans were used to confirm the direction of screw misplacement, the cranial angle, and the lateral angle. Accuracy of screw placement was determined by three independent spine surgeons blinded to the surgical approach. When two or more observers agreed on accuracy of screw placement, this was considered the consensus grade.

### Statistics analysis

Continuous data are listed as means ± SD for normal distribution, while non-continuous data were presented as numbers or ratios. The reliability of the parameters documented in two groups with SPC guide were determined by intraclass correlation coefficients (ICCs). Reliabilities > 0.75 were considered as excellent, 0.40–0.75 as fair to good, < 0.40 were characterized as poor. All statistical analyzes were calculated by SPSS Statistics 20 (IBM Corp, Armonk, New York, United States). Bias of the data conforming to normal distribution were analyzed by Bland–Altman analysis to evaluate the agreement by GraphPad Prism 8 (GraphPad Software, La Jolla, CA).

## Results

A total of 48 CBT screws were implanted into four lumbar specimens in this study, each lumbar spine specimen was implanted 12 CBT screws in the L1-S1 segment. The planning accuracy of CBT screws were evaluated by the software before the surgery, after the CBT screws were implanted according to preoperative planning, the accuracy of inserted screws was evaluated from 3D-CT reconstructions. Meanwhile, the patient demographics and parameters were shown in Table 1.

**Table 1:** Patient demographics and parameters

Patient	Gender	Age (y/o)	BMD T value	Surgical segment	Surgical approach	Screwing time (min)	Radiation dose (μSv)	Radiation time (s)
Patient 1	F	67	− 1.50	L1-S1	Planning + SPC	25	15	12
Patient 2	M	49	− 0.90	L1-S1	Planning + SPC	35	19	16
Patient 3	F	53	− 1.60	L1-S1	Planning + SPC	45	24	16
Patient 4	M	69	− 1.00	L1-S1	Planning + SPC	35	25	28

*y/o* years old, *BMD* bone mineral density, *min* minute, *μSv* microsievert, *s* second, *F* female, *M* male, *SPC* Spinous Process Clamp guide. Lumbar segments varied from the first lumbar to the first sacral segment. The values of BMD were measured using dual energy X-ray absorptiometry (DXA)

The CBT screw accuracy grades was shown in Table 2. The deviation of the 48 planning screws and 48 inserted screws was accessed based on 3D-CT reconstruction. Actually, there were 48 CBT screws were implanted in four lumbar specimens by the SPC guide method. Overall, a clinically acceptable cortical screw trajectory contains the modified Gertzbein and Robbins classification grades A and B. There were no statistically significant differences between the planning screw and inserted screw in grade A, grade B, and grade C, (p = 0.199, p = 0.714, and p = 0.242, respectively). The incidence of the proximal FJV identified in the planning screw was comparable in the inserted screw, the difference was not statistically significant (p = 0.617).

**Table 2:** Screw placement quality with modified classification of Gertzbein and Robbins

Grade	Total (n = 96)	Planning screws (n = 48)	Inserted screws (n = 48)	P value
A (n %)	85 (88.5%)	45 (93.8%)	40 (83.3%)	0.199
B (n %)	8 (8.3%)	3 (6.3%)	5 (10.4%)	0.714
C (n %)	3 (6.3%)	0	3 (6.3%)	0.242
D (n %)	0	0	0	NA
E (n %)	0	0	0	NA
A + B (n %)	93 (96.9%)	48 (100%)	45 (93.8%)	0.242
C + D + E (n %)	3 (6.3%)	0	3 (6.3%)	0.242
FJV (n %)	4 (4.2%)	1 (2.1%)	3 (6.3%)	0.617

Due to the preoperative modeling to visualized planning screw placement, the screw deviation only with grades A and B. Table 3 was just shown the screw deviation with grades B and C in axial and sagittal planes from CT reconstruction. In the preoperative planning screw trajectory, three screws were rated as grade B result of the angle limitation of the SPC guide. The deviation screws included one medial pedicle and two lateral pedicle perforations. The soft tissue remains on the spinous process led to the systematic deviation of the SPC guide in the screw placement process, five screws have been placed in the B grade, and three screws were in the C grade. The most common direction of screw misplacement was lateral pedicle (4 screws), which followed by medical pedicle (2 screws), and cephalad endplate (1 screw).

**Table 3:** Screw deviation with grade (B + C)

Deviation	Planning screws (n = 3)	Inserted screws (n = 7)
Cranial pedicle n (%)	0	0
Caudal pedicle n (%)	0	0
Cephalad endplate n (%)	0	1
Medial pedicle n (%)	1	2
Lateral pedicle n (%)	2	4
Vertebral cortex n (%)	0	0

The screw parameters were measured in planning screws and inserted screws as shown in Table 4. The lateral angle of the cortical screw trajectory was 9.2 ± 1.8° in the planning screw group versus 9.1 ± 1.7° in the inserted screw group (p = 0.662), different has not statistically significant. The cranial angle of the planning screws (22.8 ± 5.6°) was comparable with inserted screws (23.0 ± 5.1°) (p = 0.760). There were no statistically significant differences between planning screws (38.5 ± 3.9 mm) and inserted screws (39.6 ± 4.3 mm) (p = 0.272) in the distance of the screw tip.

**Table 4:** Measurements at all lumbar vertebral levels (n = 96)

Parameter	Groups	Total (n = 96)	L1 (n = 8)	L2 (n = 8)	L3 (n = 8)	L4 (n = 8)	L5 (n = 8)	S1 (n = 8)
LA (°)	Planning screws (n = 48)	9.2 ± 1.8	9.0 ± 0.8	9.9 ± 0.8	10.3 ± 0.8	11.2 ± 0.7	8.4 ± 0.8	6.2 ± 0.5
	Inserted screws (n = 48)	9.1 ± 1.7	8.6 ± 1.0	9.4 ± 0.8	10.1 ± 0.5	11.3 ± 0.8	8.6 ± 0.8	6.4 ± 0.5
	p value	0.662	0.744	0.709	0.085	0.830	0.901	0.735
	t/Z/ × 2	0.377	0.982	1.237	0.663	− 0.128	− 0.445	− 0.635
CA (°)	Planning screws (n = 48)	22.8 ± 5.6	27.7 ± 1.2	27.8 ± 3.5	24.1 ± 2.0	22.3 ± 3.7	20.5 ± 2.0	14.4 ± 4.6
	Inserted screws (n = 48)	23.0 ± 5.1	30.0 ± 1.0	27.3 ± 3.0	24.3 ± 2.0	21.2 ± 2.1	20.9 ± 2.5	15.3 ± 3.1
	p value	0.760	0.388	0.393	0.766	0.155	0.734	0.146
	t/Z/ × 2	− 0.213	− 2.765	0.328	− 0.203	0.708	− 0.347	− 0.478
DBST (mm)	Planning screws (n = 48)	38.5 ± 3.9	34.7 ± 2.9	35.4 ± 2.5	37.3 ± 1.6	38.2 ± 1.1	40.8 ± 0.9	44.8 ± 1.3
	Inserted screws (n = 48)	39.6 ± 4.3	35.5 ± 2.2	35.8 ± 1.7	37.5 ± 1.4	39.3 ± 2.2	43.7 ± 1.0	46.1 ± 1.3
	p value	0.272	0.713	0.435	0.625	0.166	0.887	0.872
	t/Z/ × 2	− 0.929	− 0.438	− 0.325	− 0.211	− 0.836	− 4.330	− 1.382

*LA* lateral angle, *CA* cranial angle, *DBST* distance of screw tip. p < 0.05 compared with the preoperative value

**Fig. 5:**
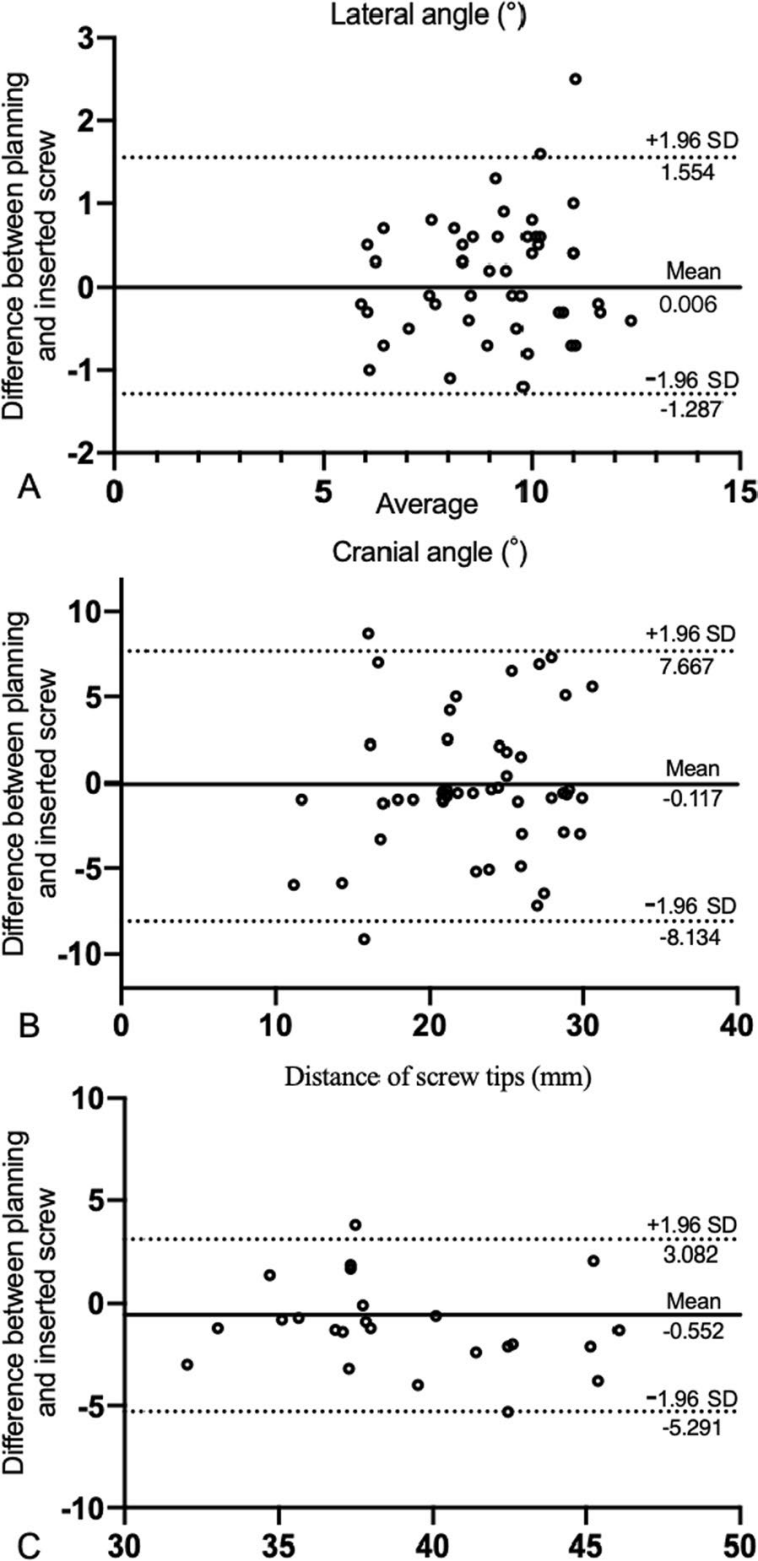
Bland–Altman Plots (95% limits of agreement) for planning screw in 3D reconstructions spine and inserted screw with SPC guide in lumbar specimens for (**A**) lateral angle, (**B**) cranial angle and (**C**) distance of screw tips

For measurements of CA, the consistency was good for ICC at 0.746 (95%CI 0.588–0.849). For LA and DBST, excellent reliabilities were demonstrated (LA: ICC 0.913, 95%CI 0.849–0.950; DOST: ICC 0.866, 95%CI 0.714–0.940) (Table 5). Bland–Altman analysis indicated good reliabilities for CA, LA and DOST (Fig. 5). The bias, 95% upper and lower limits of agreements were 0.006°, 1.554°, and − 1.287° for lateral angle and − 0.117°, 7.667° and − 7.667° for cranial angle, and − 0.552°, 3.082, and − 5.291, respectively.

**Table 5:** Results of ICCs for measurement in planned and inserted screws

Parameters	Planning screws	Inserted screws	ICC (95%CI)
CA (°)	22.80 ± 5.51	23.13 ± 5.26	0.746 (0.588–0.849)
LA (°)	9.17 ± 1.78	9.04 ± 1.68	0.913 (0.849–0.950)
DOST	38.53 ± 3.88	39.63 ± 4.34	0.866 (0.714–0.940)

*LA* lateral angle, *CA* cranial angle, *DBST* distance of screw tip

## Discussion

The cortical bone trajectory (CBT) screw technique was described to enhance the fixation strength by maximizing the screw thread contact with the cortical bone [7]. The rigid biomechanical stabilization was provided by precise placement of CBT screws to engage the pedicle and vertebral body through the four-point cortical bone, including starting point, pedicle medial wall, pedicle lateral wall, and vertebrae cortex or cephalad endplate [8]. Numerous cadaveric studies have demonstrated the accuracy of the cortical screw was improved by using 3D printed guide templates in lumbar spine surgery [3, 9, 10]. Other scholars have been reported 3D navigation and robot-assisted surgery systems have been established to improve the accuracy and safety of screw placement [4, 5].

However, there were some disadvantages of those novel screw insertion technology. The paraspinal muscles, ligaments, and facet joint capsule needed to remove for the 3D printed guide template close contact with the spinous process and lamina [3]. The high costs of the guide plate and the transportation time are both cost of factors have to be considered. On the other hand, intraoperative computed tomography is required for both 3D navigation and robotic assistance systems, resulting in increased radiation exposure for operating room staff and patients. Hence, we invented the SPC guide as a reusable, convenient, and fast guide CBT screw placement device.

This study initially described the accuracy of a cortical screw inserting utilized the SPC guide. A total of four lumbar spine specimens with L1-S1 vertebrae contained twelve screws implanted in each specimen, for a total of 48 screws. Our results demonstrated that application of the SPC guide during cortical screw placement could provide reliable execution of preoperative screw planning and accuracy and safety were improved significantly. The clinically acceptable screw placement (corresponding to grade A and grade B screws) for the planning screw (93.8%) was comparable to the accuracy of the inserted screw (83.3%) (p = 0.242). The incidence of proximal facet joint violation (FJV) for planning screws (1.2%) was comparable with inserted screws (6.3%) (p = 0.617), which indicated that the SPC guide was fixed on the spinous process firmly during the screw implant progress.

The deviation of the planning screw and the inserted screw were measured from the superimposed 3D reconstruction of the screw and vertebrae pre- and postoperative. The accuracy of the CBT screw placement was graded according to the modified method of Gertzbein and Robbins, (Grade A, no perforation; Grade B, 0–2 mm; Grade C, 2–4 mm; Grade D, 4–6 mm; Grade E > 6 mm). Since the preoperative screw planning was carried out in the Mimics^®^ (Materialise, Leuven, Belgium) for 3D modeling of the spine, planning the SPC guide to implant the screw under the condition of the whole spine visualization can avoid the screw thread penetrating the outer cortex of the pedicle vertebral body as precise as possible. However, in this study, there were three planning screws record as grade B, the possible explanation was that the planned K-wire inserted the vertebrae through the SPC guide was much thinner than the screw model. Despite the pedicle along the cortical trajectory could insert a planned K-wire, it was still too narrow to insert a CBT screw, and the plan was used to minimize the perforated incidence. The explanation of inserted screw placement as grade B or C was that the putation disadvantage of a K-wire-based guide through SPC guide has a high risk for bowing because its diameter was too thin to keep straight.

The direction of the cortical screw perforation including caudal pedicle, medial pedicle anterior and lateral walls of the vertebral body, and cephalad endplate results in nerve root irritation, spinal cord injuries, disc degeneration, vascular, and biomechanical defect [11–13]. In the axial plane, there were several potential reasons to explain the screw deviation. First, confirming the clamp at the head of the SPC guide rode on the spinous process appositely of the surgical vertebral body was a crucial step to achieve accurate navigation, and adjusting the knob height is one of the key factors in determining the lateral angle. The knob was closer to the spinous process, increasing the lateral angle of the screw due to the obstruction of the spinous process. The worst situation was that inadequate trochoid motion during the screwing process can cause pars or pedicle fracture, and caudal or medial deviation can threaten neural tissue [14, 15]. The knob can be adjusted away from the spinous process to provide adequate space between the screw tail and the spinous process at the expense of the lateral angle. Second, while making an initial screw hole, the tip of the K-wire is likely slipping lateral side because on the isthmus ridge is the entry point. That is why lateral perforation was the most common direction in inserted screws.

In the sagittal plane, the caudal deviation can jeopardize neural tissue, cranial deviation causes proximal facet joint violation (FJV), and cephalad endplate led to disc degeneration. The present study demonstrated that the most common deviation was cephalad endplate followed by caudal perforation. The anchor site of the SPC guider with clamp was planned before surgery and pinched into the spinous process, the cranial angle of the column of the guide was adjusted based on preoperative planning, a K-wire was stabbed into the spinous process through the column and checked with lateral imaging at last. During this process, there will be an acceptable slight displacement of the clip and the spinous process, and the angle of the caudal inclination of the post will increase due to the influence of gravity, which can cause cephalad endplate perforation. With the increased caudal inclination of the SPC guide, the guided screw is more likely to involve the proximal facet joint.

Parameters of any deviation of inserted screws tips from planned screws were measured through the preoperative and postoperative 3D-CT reconstructed vertebrae and screws superimposed. Our results demonstrated no statistically significant difference between the distance of planning screws tip and inserted screws tip. The tips of the screws were selected as the cortical screw deviation parameter which was measured with 3D models reconstructed from CT scan. Due to the screw entry point is the anchor for screwing the screw, the position is constant with a slight deviation. The deviation of the screw direction during the insertion process will eventually be enlarged at the screw tip, so the screw tip is the best indicator for measuring the screw deviation.

The present study has some limitations. First, the SPC guide was adjusted manually based on the preoperative cortical screw parameter, which enlarged the deviation during the insertion of screws. Second, the small sample size of this study limits the evaluation of screw accuracy, and the conclusions drawn lack credibility and representativeness. Third, further clinical studies are needed to evaluate the safety, efficacy of screw insertion with the SPC guide. Finally, when forced to adjust the SPC guide may cause spinous process fractures.

## Conclusions

In conclusion, this cadaveric study shows that the influence of gravity on the SPC guide can lead to an increase in the cranial angle of the screw. Although it may increase the incidence of penetration of the cranial wall of the pedicle and the cephalad endplate of the vertebral body, it will reduce the penetration of the caudal pedicle wall, which enable lead to serious neurological complications. Restricted by the spinous process, the screw planned by the SPC navigator is lateral and has a larger abduction angle. Although it will increase the damage of the pedicle or the lateral wall of the vertebral and also reduce the damage of the medial wall that leads to nerve root damage. The SPC guide screws placement accurately and reduces severe deviations in important directions. More clinical results are needed to confirm the imaging and clinical effects in the future.

## Data Availability

The data used and analyzed in this study are included in the article or are available from the corresponding and first authors upon reasonable request.
